# Hypothalamic orexigenic and anorexigenic neuropeptides in the rotenone model of Parkinson’s disease

**DOI:** 10.1038/s41598-026-51774-7

**Published:** 2026-05-04

**Authors:** Zsombor Márton, Bence Pytel, Dávid Kovács, Máté Szabó, Zsófia Havasi, Gergely Berta, József Farkas, László Ákos Kovács, Nóra Füredi, Viktória Kormos, Balázs Gaszner

**Affiliations:** 1 Department of Anatomy, Medical School, University of Pécs, Szigeti út 12, Pecs, 7624 Hungary; 2 Research Group for Mood Disorders, Centre for Neuroscience, Medical School, University of Pécs, Szigeti út 12, Pecs, 7624 Hungary; 3 Department of Medical Biology, Medical School, University of Pécs, Szigeti út 12, Pecs, 7624 Hungary; 4 Department of Pharmacology and Pharmacotherapy, Medical School, University of Pécs, Szigeti út 12, Pecs, 7624 Hungary

**Keywords:** Body weight, Levodopa, Fluoxetine, NPY, Orexin-1, α-MSH, CART, Diseases, Drug discovery, Neurology, Neuroscience

## Abstract

Beyond depression and anxiety, non-motor symptoms of Parkinson’s disease (PD) also include body weight changes with an unclear neurobiological background. Here, we aimed to characterize the functional-morphological changes of the main orexigenic and anorexigenic hypothalamic peptidergic systems in the rotenone model of PD in male rats. We also tested their response to benserazide/levodopa (B/L) anti-PD therapy alone or in combination with antidepressant (fluoxetine) medication. In the model, anhedonia and compromised motor coordination were observed in sucrose preference and rotarod tests respectively, with an acceptable therapeutic response. RNAscope in situ hybridization, combined with immunofluorescence, revealed that rotenone did not cause significant peptidergic neuron loss in the hypothalamus but reduced the expression of hypocretin and proopiomelanocortin mRNAs, in line with reduced orexin-1 and α-melanocyte-stimulating hormone immunoreactivities. The expression of neuropeptide y and cocaine- and amphetamine-regulated transcript was downregulated only at the mRNA level. B/L alone or in combination with fluoxetine failed to reverse these functional-morphological changes. Our findings suggest complex functional alterations in both orexinergic and anorexigenic peptidergic systems in the rat model of PD that are not reversed by anti-PD and antidepressant administration.

**Supplementary Information:**

The online version contains supplementary material available at 10.1038/s41598-026-51774-7.

## Introduction

Parkinson’s disease (PD) involves both motor and non-motor symptoms. Rigidity, tremor, and bradykinesia characterize the motor deficits that are attributed to the neurodegeneration of dopaminergic neurons in the substantia nigra^1,2^. Non-motor symptoms span a wide spectrum. They include neuropsychiatric symptoms like depression, anxiety, and cognitive difficulties. Autonomic disturbances such as orthostatic hypotension, constipation, and urogenital dysfunction, along with sleep disorders and impairments in the sense of smell may also occur^3^. These signs frequently manifest earlier than motor impairments^4^. Moreover, patients often report that these symptoms have a greater impact on their quality of life compared to motor-related issues^5^.

Non-motor symptoms also include changes to the body weight that might appear both as weight gain or loss^6^. Weight loss in PD can be attributed to diminished food intake caused by impaired olfactory and gustatory functions, the restrictive impact of motor symptoms, and elevated energy expenditure resulting from tremor, dyskinesia, and rigidity^7^. Conversely, weight gain may arise from improved motor capabilities and enhanced swallowing facilitated by medication, or from compulsive eating behaviors as a side effect of treatment^8^. Sharma and colleagues (2017) identified two distinct weight-related phenotypes in PD. Phenotype A is characterized by significant olfactory loss, higher baseline body weight, and progressive weight reduction over the disease course. In contrast, phenotype B involves moderate olfactory impairment, lower initial body weight, and subsequent stabilization or even an increase in body weight during disease progression^9^.

Another aspect of the body weight change in PD is the regulation of homeostatic food intake, controlled by four key neuropeptides produced by hypothalamic neurons.

Neuropeptide Y (NPY) is synthesized by cells situated in the medial division of the hypothalamic arcuate nucleus (ARC)^10,11^. The release of this neuropeptide is promoted by orexinergic signals, including the secretion of ghrelin or reduced production of leptin. Meanwhile, it is suppressed by anorexigenic factors such as higher levels of glucose, leptin, or insulin^10–12^. NPY/ARC neurons project to the lateral hypothalamus (LH), where they stimulate the production of orexinergic peptides like orexin-1 (also referred to as hypocretin)^10^. Orexin-1 primarily targets two G protein-coupled receptors, OX1 and OX2. Orexin-producing neurons in the hypothalamus project to dopaminergic cells in the ventral tegmental area (VTA) and modulate via the mesolimbic pathways the nucleus accumbens. Through these circuits, they play a key role in the hedonic regulation of food intake. In addition, orexin signaling promotes wakefulness and increases physical activity. Therefore, a decrease in orexin levels reduces appetite and subsequent weight loss^11,13^.

In the lateral division of ARC, a distinct group of neurons produces the pro-opiomelanocortin (*Pomc*) mRNA. The POMC protein product is cleaved into several neuropeptides^14^ including alpha-melanocyte-stimulating hormone (α-MSH)^12^. α-MSH targets melanocortin receptors in the central nervous system, including MC3R and MC4R. MC3R controls feeding efficiency, while MC4R regulates food intake and energy metabolism^15^. POMC/ARC cells are activated by anorexigenic signals, such as leptin or glucose release, leading to a decrease in food intake and an increase in energy expenditure. The POMC/ARC neurons co-express another anorexigenic neuropeptide, cocaine- and amphetamine-regulated transcript (CART). CART targets the GPR160 orphan receptor in the nucleus of the solitary tract and exerts its anorexigenic effect via vagal afferents. It influences the activity of appetite-related regions adjacent to the area postrema, leading to a reduction in food intake^16^. Additionally, POMC/CART/ARC neurons project to the paraventricular nucleus of the hypothalamus, where they stimulate the release of anorexigenic neuropeptides like corticotropin-releasing hormone (CRH). This, in turn, reduces dopamine release associated with rewarding behavior, ultimately leading to decreased appetite^10,17^.

It has been reported that patients with Parkinson’s disease often present with symptoms that may reflect underlying hypothalamic dysfunction, such as thermoregulatory dysfunction^18^, REM sleep disturbances^19^, and alterations in body weight^20^. Furthermore, Lewy body deposition has been identified across all hypothalamic nuclei in Parkinson’s disease^21^, supporting the presence of underlying hypothalamic pathology.

Previous research has shown reduced NPY peptide level in cerebrospinal fluid (CSF) samples from Parkinson’s disease patients, while another research group found elevated NPY mRNA level in the caudate nucleus, putamen, and nucleus accumbens, which may also affect weight change^22^. In PD, studies of human samples have documented a selective loss of orexin-producing neurons in the hypothalamus, which has been associated with disease progression. Additionally, decreased levels of orexin-1 have been observed in postmortem CSF and prefrontal cortex samples. Consequently, diminished orexin production may significantly contribute to the energy imbalance and weight fluctuations observed in PD^23–25^. It is also well established that reduced orexin-1 levels can lead to sleep disturbances. This may contribute to the development of REM sleep abnormalities observed in Parkinson’s disease^26,27^.

Although studies on α-MSH levels in neurodegenerative diseases remain limited, available evidence indicates a significantly increased CSF concentrations in PD^28^. Consistent with this, a study in Alzheimer’s disease reported a negative correlation between α-MSH and homovanillic acid levels, further suggesting a potential elevation of α-MSH in Parkinson’s disease^29^.

In the rotenone model of Parkinson’s disease in rats, REM sleep disturbances^30^ and disruptions in bodyweight gain^31^, metabolic peptide balance^32^ have been observed. These alterations suggest hypothalamic dysfunction and the possible recruitment of hypothalamic peptidergic systems in the model.

In the past few years, increasing attention has been given to non-motor symptoms in PD. However, the background of an altered energetic state in PD and the potential contribution of the main orexigenic and anorexigenic peptidergic systems have not been systematically investigated. Therefore, we decided to examine the main orexigenic and anorexigenic hypothalamic factors at mRNA and peptide level in the rotenone model of PD, in the rat. Because the effect of the commonly applied anti-Parkinson and antidepressant medications on these peptidergic systems in PD models has not been examined either, we supplemented our model with groups that underwent benserazide/levodopa (B/L) therapy alone or in combination with fluoxetine. Behavioral tools were applied to prove the motor and non-motor symptoms of PD and to validate the efficacy of the pharmacotherapy. We hypothesized that the mRNA expression and peptide content of hypothalamic orexigenic (orexin-1 and α-MSH) and anorexigenic (NPY and CART) peptidergic neurons would be affected in the PD model. Due to frequent weight loss in PD, we expected downregulation of orexinergic and upregulation of anorexinergic neuropeptides. Based on our hypothesis, the drug therapies used are likely to have limited efficacy in reversing these effects. RNAscope in situ hybridization combined with immunofluorescence was applied to characterize the functional-morphological changes in the POMC/CART as well as NPY neurons of the ARC and orexin-1 neurons of the LH.

## Methods

### Animals

In-house bred male Wistar rats (breeding pairs obtained from Animalab Kft., Vác, Hungary) were housed in pairs in standard-sized plastic cages (38 × 22 × 15 cm) under controlled conditions with 60% humidity and a temperature range of 23–26 °C. A 12-hour light phase was maintained between 6:00 am and 6:00 pm. Animals had free access to water and food. The experiments were approved by the Animal Welfare Committee of Pécs University, the National Scientific Ethical Committee on Animal Experimentation in Hungary, and the National Food Chain Safety Office in Hungary (license No: BA02/2000-83/2022). All in vivo tests and experiments were performed in accordance with the ARRIVE guidelines and other relevant regulations.

### Systemic rotenone treatment model of PD and pharmacotherapy

Rats were 6-month-old when randomly assigned randomly into four groups. Their body weight was measured every week before the first injection of the day. A PD-like condition was induced in 6 weeks by 1.5 mg/kg/day subcutaneous (s.c.) rotenone (R) treatment (*n* = 45), along with its solvent 20 µL/kg/day dimethyl sulfoxide (DMSO) (Fisher Scientific, Loughborough, UK) and 1 mL/kg sterile sunflower oil (1 ml/kg, 8000-21-6, Molar Chemicals Kft., Halásztelek, Hungary). The control group (*n* = 10) received only DMSO and sterile sunflower oil in the same dose as the neurotoxin-treated group. Starting from the fourth week, one part of the neurotoxin-treated group (*n* = 11) received no further medication, another part (*n* = 11) underwent 10 mg/kg levodopa therapy (PHR1271, Merck) with 2.5 mg/kg benserazide-hydrochloride (Merck, Darmstadt, Germany) dissolved in 0.5 ml 0.9 M NaCl saline twice a day [^33^, Pytel et al. (submitted)], and the final subgroup (*n* = 11) received fluoxetine (F) (s.c. 5 mg/kg/day) combined with the B/L medication as described above. Animals in both the unmedicated and control groups were administered also with 0.5 ml of s.c. saline injections (Table 1). The efficacy of our animal model was verified in the rotarod test, which assesses motor symptoms. Non-motor symptoms such as anxiety and depression-like behavior were examined in open field and in sucrose preference tests, respectively. For further details, such as assessment of dopaminergic neuron loss, exclusion criteria and humane end point, we refer to our recently published works [^33^, Pytel et al. (submitted)]. In this study, four rats were removed from the experiment due to humane end point, and in three additional rotenone-treated rats were excluded because the dopaminergic neuron loss was not satisfactory^33^. Also due to technical reasons (e.g. loss or damage of sections during histological procedure), in some morphological assessments in the rotenone group we have less animals (*n* = 5–6) than in the other groups (*n* = 8–11). However, this sample size is still normal in functional-morphological tests.

**Table 1:** Table of doses. The oil (control) group received solvent injections: sunflower oil (1 ml/kg) with dimethyl sulfoxide (DMSO) and saline. Besides a rotenone (R)-treated group, we created a group that received R also benserazide/levodopa (R + B/L) treatment, moreover a group (R + B/L + F) that received also fluoxetine (F) injections.

	Oil	R	R + B/L	R + B/L + F
Rotenone Sunflower oil DMSO	- 1 ml/kg 20 µL/kg	1.5 mg/kg/day 1 ml/kg 20 µL/kg	1.5 mg/kg 1 ml/kg 20 µL/kg	1.5 mg/kg/day 1 ml/kg 20 µL/kg/day
Levodopa Benserazide-hydrochloride NaCl saline	- - 2 × 0.5 ml 0.9 M	- - 2 × 0.5 ml 0.9 M	2 × 10 mg/kg 2 × 2.5 mg/kg 2 × 0.5 ml 0.9 M	2 × 10 mg/kg 2 × 2.5 mg/kg 2 × 0.5 ml 0.9 M
Fluoxetine NaCl saline	- 0.5 ml 0.9 M	- 0.5 ml 0.9 M	- 0.5 ml 0.9 M	5 mg/kg/day 0.5 ml 0.9 M

### Tissue and sample preparation

At the end of week 6, rats were anesthetized with an intraperitoneal overdose of urethane (2 g/kg, Merck KGaA, Darmstadt, Germany). The thoracic cavity was opened, the ascending aorta was cannulated, and the descending aorta was clipped. Rats were perfused with 50 ml of 0.1 M sodium phosphate-buffered physiological saline (PBS, pH 7.4) followed by 250 ml of 4% paraformaldehyde (PFA) solution in Millonig buffer in 20 min. Subsequently, brains were removed and post-fixed in the above-described fixative at 4 °C until sectioning. Additionally, both adrenal glands and the thymus were excised and weighed.

Brains were sectioned using a vibratome to obtain 30 μm free-floating coronal sections. For this study, coronal hypothalamic sections containing the ARC and LH were manually selected between the planes − 1.80 mm and − 3.60 mm to Bregma, based on the rat brain atlas^34^. Sections were then stored in an antifreeze solution at -20 °C until further use.

### RNAscope in situ hybridization combined with immunofluorescence

Four representative sections *per* animal were selected from the ARC and LH regions respectively, for each staining. The sections were subjected to a modified RNAscope pretreatment protocol tailored for 30 μm PFA-fixed sections as we recently published^35,36^. Subsequent steps of the RNAscope procedure were carried out in accordance with the manufacturer’s guidelines (Advanced Cell Diagnostics, Newark, CA, USA, ACD).

The following probes were purchased from ACD to detect the respective mRNAs: rat *Hcrt* (Cat No: 401151-C3), rat *Pomc* (Cat No: 318511-C2), rat *Npy* (Cat No: 450971-C2) mouse *Cart* (Cat No: 432001-C2). In a pilot experiment, we confirmed that the mouse *Cart* probe detects *Cart* mRNA in rat tissue as well. This was expected based on the 97,1% amino acid sequence overlap between mouse and rat CART^37^.

Subsequent to channel development, preparations were rinsed with PBS and we applied primary antibodies to detect the respective peptide products of the above listed mRNAs. We incubated the preparations in the following antisera overnight at room temperature: polyclonal goat anti-orexin-1 (1:2000, Cat No: sc-8070, Santa Cruz, RRID: AB_653610), polyclonal rabbit anti-alpha-MSH (1:5000, Cat No: T-4434, Peninsula Laboratories International and BMA Biomedicals, Augst, Switzerland, RRID: AB_518449), polyclonal rabbit anti-CART (1:5000, Cat No: H-003-62, Phoenix Europe GmbH, Karlsruhe, Germany, RRID: AB_2313614), polyclonal sheep anti-NPY (1:48:000, FJL #14/3A, gift by Dr. Istvan Merchenthaler, University of Maryland, Baltimore MD, USA). After PBS washes, the respective fluorophore-conjugated secondary antisera (all from Jackson Immunoresearch Europe Ltd., Cambridgeshire, UK) diluted in PBS with 2% normal donkey serum, (NDS, Cat No: 017-000-001, RRID: AB_2337254) were applied for 3 h: Alexa 488-conjugated donkey anti-goat (1:500, Cat No: 705-545-147, RRID: AB_2336933B), Cyanine 3 (Cy3)-conjugated donkey anti-rabbit (1:500, Cat No: 712-165-153, RRID: AB_2340667) and Alexa Fluor 647-conjugated donkey anti-sheep (1:500, Cat No: 713-605-003, RRID: AB_2340750). Finally, after washes, sections were counterstained with 4′,6-diamidino-2-phenylindole (DAPI, ACD) and covered with anti-fade medium.

Triplex positive (Cat No: 320891, ACD) and negative control (Cat No: 320871, ACD) probes were applied to test the sensitivity of the RNAscope assay on randomly selected hypothalamic sections. In preparations treated with positive control probes obvious cytoplasmic fluorescence was recognizable. In the negative control, we did not see signal dots.

### Double-label immunofluorescence

Further free-floating hypothalamus sections were subjected to double-label immunofluorescence as recently published^31^. Briefly, after several washes in PBS, epitope retrieval was applied in citrate buffer at 90 °C for 10 min. Then, sections were permeabilized in 0.5% Triton x 100 solution for 30 min, blocked in 2% NDS for 60 min. After PBS rinses, sections were incubated overnight at room temperature in a cocktail of primary antibodies with mouse monoclonal anti-FOSB (1:1500, Cat No: ab11959, Abcam, RRID: AB_298732) and goat anti-orexin-1 (1:2000, Cat No: sc-8070, Santa Cruz, RRID: AB_653610). Following PBS washes, Cy3-conjugated donkey anti-mouse (diluted to 1:500 in PBS containing 2% NDS, Cat No: 715-095-151, RRID: AB_2335588) and Alexa 488-conjugated donkey anti goat (diluted to 1:500 in PBS containing 2% NDS, Cat No: 705-545-147, RRID: AB_2336933B) antisera were used for 3 h. After washes, sections were mounted on gelatin-coated slides, air dried and covered with glycerol-PBS (1:1) solution.

### Antibody controls

The goat orexin-1 antibody targets the C-terminus of human orexin-A. The specificity of the orexin-1 antibody (Cat No: sc-8070, Santa Cruz, RRID: AB_653610) has been confirmed in rat brain by Western blot (https://www.scbt.com/p/orexin-a-antibody-c-19 accessed on 21st April 2026) and validated by multiple research groups^38,39^. The polyclonal α-MSH antibody (Peninsula Laboratories, Cat No: T-4434, RRID: AB_518449) was generated in the rabbit using the synthetic peptide Ac-Ser-Tyr-Ser-Nle-Glu-His-D-Phe-Arg-Trp-Gly-LysPro-Val-NH2 and its specificity has been tested in rat tissues in our laboratory^26^. The supplier published that the serum shows negligible cross reactivity with related neuropeptides (https://www.bma.ch/files/product/T-4434.pdf accessed on 21st April 2026) The polyclonal anti-CART antibody (Phoenix Pharmaceuticals, Cat No: H-003-62, RRID: AB_2313614) was generated in the rabbit against rat CART 55–102 peptide and its specificity was also confirmed in the rat^40^. The specificity and sensitivity of the polyclonal sheep NPY antibody (FJL #14/3A, gift from Dr. Istvan Merchenthaler) has been tested in the rat brain by our laboratory^39^ and other research groups also^41^. The monoclonal FOSB antibody, produced in mice, has undergone specificity testing via Western blot (https://www.abcam.com/en-us/products/primary-antibodies/fos-b-antibody-83b1138-ab11959 accessed on 21st April 2026) and has been effectively utilized by our research team^42^. In this study, the labeling was entirely eliminated in all cases when primary or secondary antisera were omitted or replaced with corresponding normal sera.

### Microscopy, digitalization and morphometry

A confocal laser scanning microscope (Olympus FluoView 1000, Olympus Europe, Hamburg, Germany) was used to analyze the fluorescence-labeled sections. Images were captured at a resolution of 1024 × 1024 pixels using a confocal aperture of 80 μm and a 40x objective lens. Specific lasers were used to excite the fluorophores as follows: DAPI: excitation at 405 nm, detection between 425 nm and 475 nm, Alexa 488 excitation at 488 nm, detection between 500 nm and 530 nm; Cy3: excitation at 543 nm, detection between 560 nm and 620 nm; Alexa 647: excitation at 633 nm, detection between 640 and 670 nm. Virtual colors were assigned to channels for visualization purposes: blue for DAPI, green for Alexa 488, red for Cy3, white for Alexa 647. Using the microscope’s proprietary software, images for each fluorescent channel were acquired sequentially and then merged to provide composite visualizations as needed.

Morphometric measurements, including cell counting and densitometry, were manually performed using ImageJ software version 1.54j by an observer who was not informed about the identity of images and preparations. For each animal and brain area, four representative sections were evaluated. To determine the specific signal density (SSD), we selected 5 representative cells per image crossected through their cell nucleus. The immunofluorescence density of cells was measured in manually circumscribed cytoplasmic areas by using the freehand selection tool. The fluorescence intensity of NPY immunoreactivity was measured upon a precise manual selection of five well-trackable axon segments per section. The fluorescence intensity signal measured in the cell bodies or axons was adjusted by subtracting background signal values measured outside the selected cells or axons in areas were no specific signal was recognizable. This calculation provided the SSD values that were averaged for each image of a section. Finally, the mean SSD values of four sections of the same animal was calculated representing one animal in the statistical analysis^33,43^. Cell counts were manually determined in images of 4 sections per animal in a representative 300 × 300 μm area of the examined ARC and LH regions. These numbers were averaged as described above for the SSD values.

### Statistics

The normality of the data distribution and homogeneity of variance were assessed through Kolmogorov-Smirnov, Shapiro-Wilk, and Bartlett tests. To control for potential baseline differences between groups and facilitate reliable correlation analysis, the raw values were converted to Z-score using the formula: Z = (X - µ)/σ, where X represents the raw data for each animal, µ is the mean, and σ is the standard deviation of the control group. One-way analysis of variance (ANOVA) was performed and the statistical results were summarized in Supplementary Table 1. In case of positive outcome in the ANOVA, the pairwise comparisons of groups was done with Tukey’s post hoc test, which is specifically recommended for multiple comparisons after ANOVA^44^. Statistical correlations between datasets were also examined, by Spearman test. Results were shown as a heat map with the corresponding rho values. The corresponding p values are summarized in Supplementary Table 2. The level of significance was set at α = 5%, in all cases.

## Results

In full agreement with our recent works^31,33^, behavioral tests supported the efficacy of the rotenone model. In the rotenone-treated group, a significant deficit in motor coordination was observed compared to the control group (ANOVA: F(3,44) = 11.26; *p* < 10^−4^, oil vs. R, Tukey’s post hoc test: *p* < 10^−4^). This impairment was reversed both by antiparkinsonian therapy alone (R vs. R + B/L, Tukey’s post hoc test: *p* = 0.004) and by the combined therapy (R vs. R + B/L + F, Tukey’s post hoc test: *p* < 10^−4^). In the sucrose preference test, increased anhedonia was observed in the neurotoxin-treated group (ANOVA: F(3,27) = 4.549; *p* = 0.01; oil vs. R, Tukey’s post hoc test: *p* = 0.014). This impairment was not reversed by antiparkinsonian therapy alone (R vs. R + B/L, Tukey’s post hoc test: *p* = 0.31), whereas the combined therapy proved effective (R vs. R + B/L + F, Tukey’s post hoc test: *p* = 0.035). [^33^, Pytel et al. (submitted)]. The link between deterioration of motor skills and mood status was also supported by the correlation between these variables (Spearman test: ρ = 0,48; *p* = 0.0076).

**Fig. 1:**
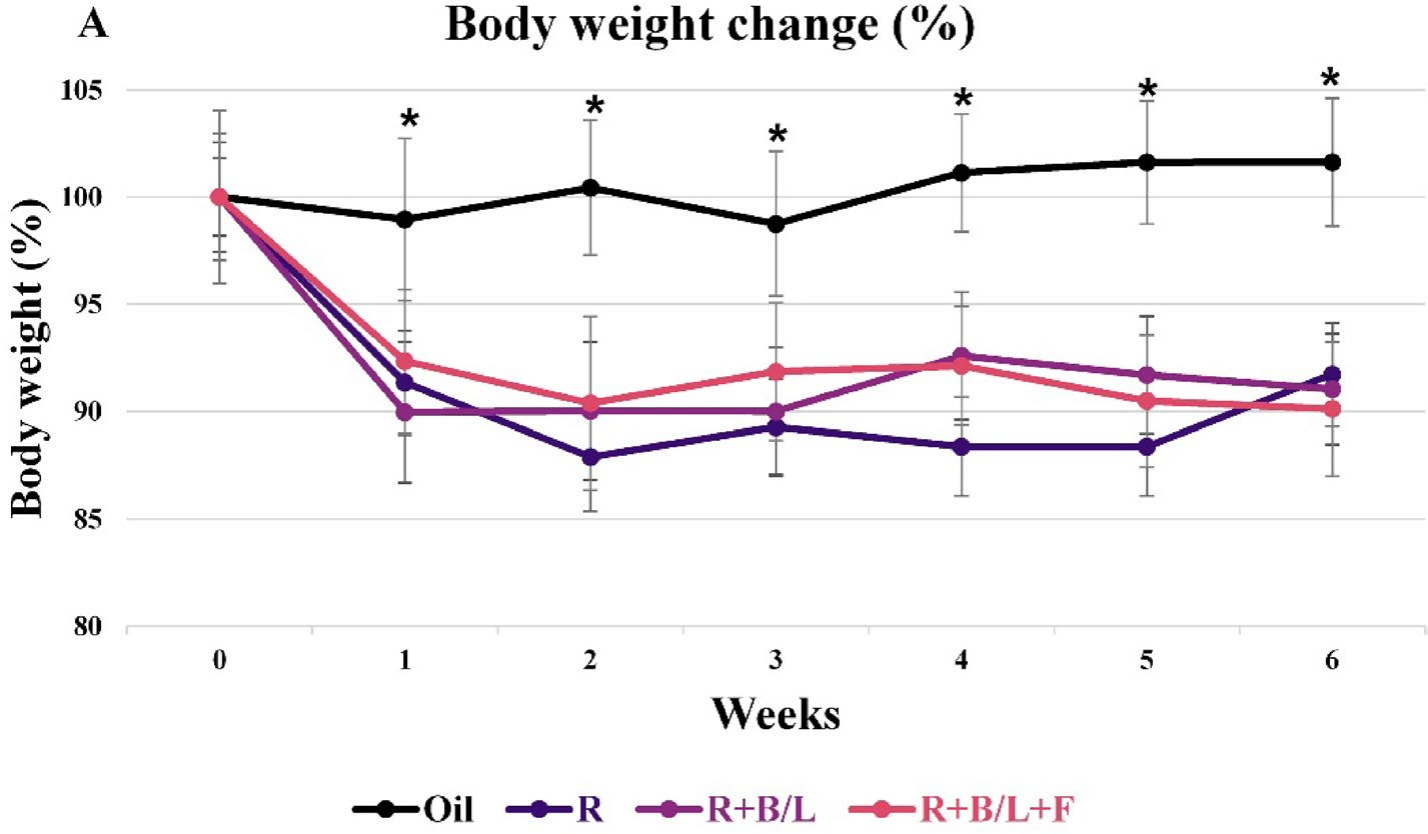
Body weight changes. Diagram A shows the body weight curves of groups in the 6 weeks period of rotenone administration. The y-axis shows percentage relative to baseline; the x-axis indicates time in weeks. Oil: vehicle-treated control. R: rotenone-injected, untreated group. R + B/L: rotenone-treated group benserazide/levodopa (B/L) therapy. R + B/L + F: rotenone-treated group subjected to B/L and fluoxetine (F) treatment. *p < 0.05; Tukey’s *post hoc* upon one-way ANOVA.

During the in vivo experimental phase, the body weight of the animals was measured weekly. No significant difference (ANOVA: F(3,41) = 2.314; *p* = 0.0904) was observed in baseline body weight between the groups (initial bodyweights: Oil: 672 ± 25.4 g R: 666.92 ± 12.37 R + B/L: 702.73 ± 18.74 R + B/L:633.64 ± 17.89; Suppl. Fig. 1). The change in body weight was calculated and monitored as a percentage of the baseline value. Over the six-week period, the neurotoxin-treated group showed a significant (ANOVA: F (3,32) = 4.86; *p* = 0.0056) reduction in body weight compared to the control group (Fig. 1 and Suppl. Table 1). This weight loss was not influenced by the administered drug treatments.

**Fig. 2:**
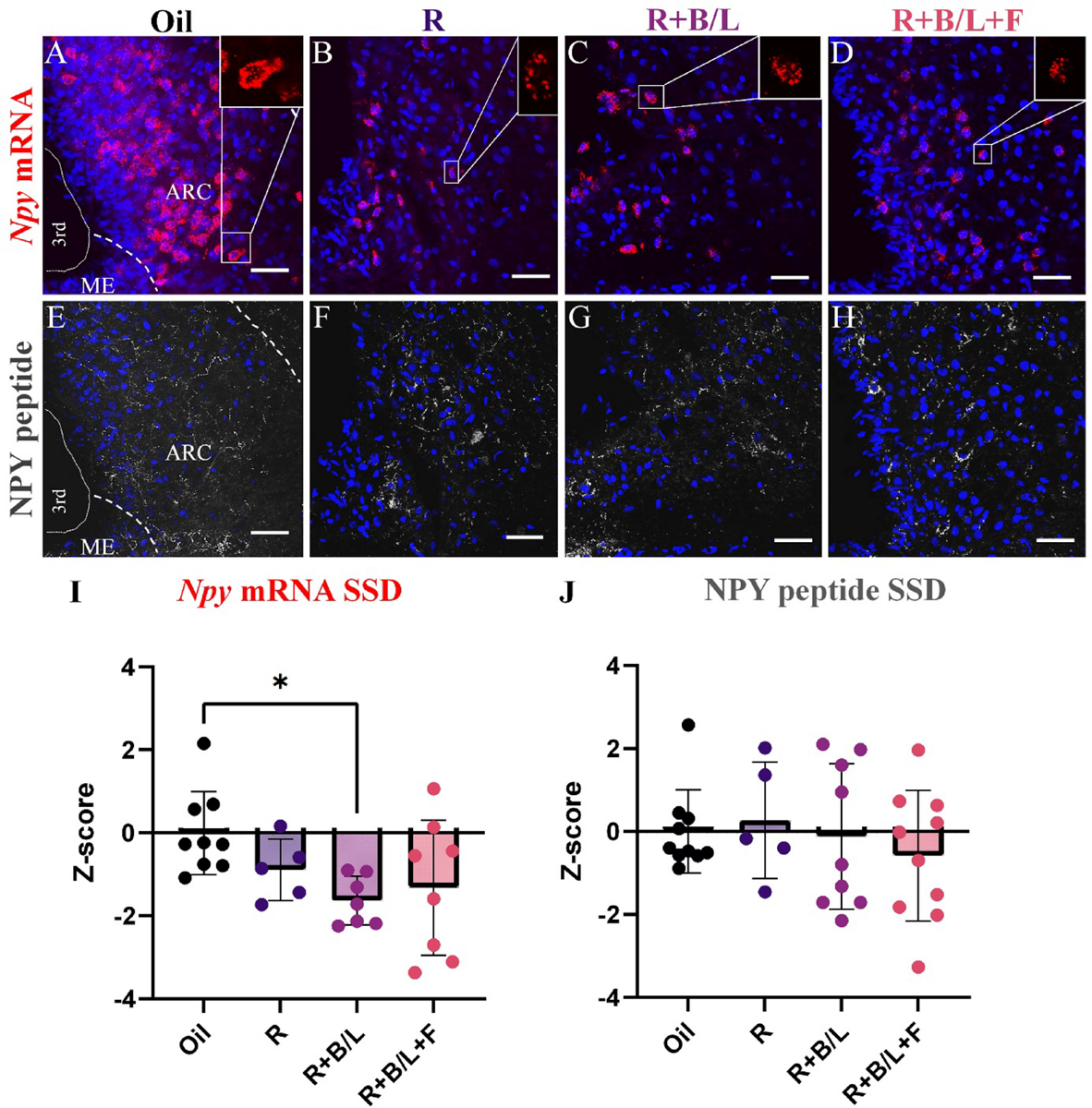
Neuropeptide Y mRNA (*Npy)* expression and NPY peptide immunoreactivity in the arcuate nucleus (ARC) in the rotenone model. Representative images show *Npy* mRNA (**A**–**D**, in red) expression and NPY peptide (**E**–**H**, white) immunofluorescence in vehicle (**A** and **D**: oil), (**B** and **F**) rotenone (R)-injected rats. Panel C and G represent a R-treated rat that received benserazide/levodopa therapy also (R + B/L). Panel D and H show representative images from a sample of a rat that received besides R + B/L therapy fluoxetine injections also (R + B/L + F). In panels A-H blue color refers to 4′,6-diamidino-2-phenylindole (DAPI) nuclear counterstaining. Scale bar: 50 μm. Panel (**I**) illustrates the comparison of *Npy* mRNA specific signal density (SSD) in the ARC. Histogram (**J**) provides the comparison of NPY immunoreactivity in the nerve fibers of the ARC. *p < 0.05; *n* = 5–9; one-way ANOVA with Tukey’s *post hoc* test. 3rd: third ventricle; ME: median eminence.

In the ARC, we successfully conducted in situ hybridization for *Npy* mRNA combined with immunofluorescence for NPY neuropeptide. In line with our expectation, we detected confluent cytoplasmic *Npy* signal puncta in the ARC. This labeling was intense enough to perform a reliable cell counting. The comparison of the cell counts in our experimental groups revealed that the number of *Npy* mRNA-expressing cells did not change (Fig. 2A–D, I, ANOVA: F (3,32) = 2.19; *p* = 0.0546). The *Npy* mRNA expression was semi-quantified in the somata (Fig. 2A–D, I, ANOVA: F (3.25) = 3.36; *p* = 0.0346). Although the *post hoc* comparisons showed that the *Npy* mRNA expression was reduced by antiparkinsonian therapy (Fig. 2A–D, I, ANOVA: F (3,25) = 3.3602; *p* = 0.0346, Tukey’s *post hoc*: *p* = 0.035), no significant decrease was observed in the other two groups. We also found a correlation between *Npy* mRNA expression and the magnitude of body weight change in our rats (Spearman test: ρ = 0,49; *p* = 0.006).

The NPY immunoreactivity was localized to the nerve fibers, while it was nearly undetectable in the somata. Therefore, the NPY peptide immunosignal was measured in the nerve fibers within the ARC. Neither the rotenone treatment nor the anti-parkinsonian therapy or additional fluoxetine administration affected the NPY immunosignal in the ARC (Fig. 2E–H, J, ANOVA: F (3,32) = 0.469; *p* = 0.706).

**Fig. 3:**
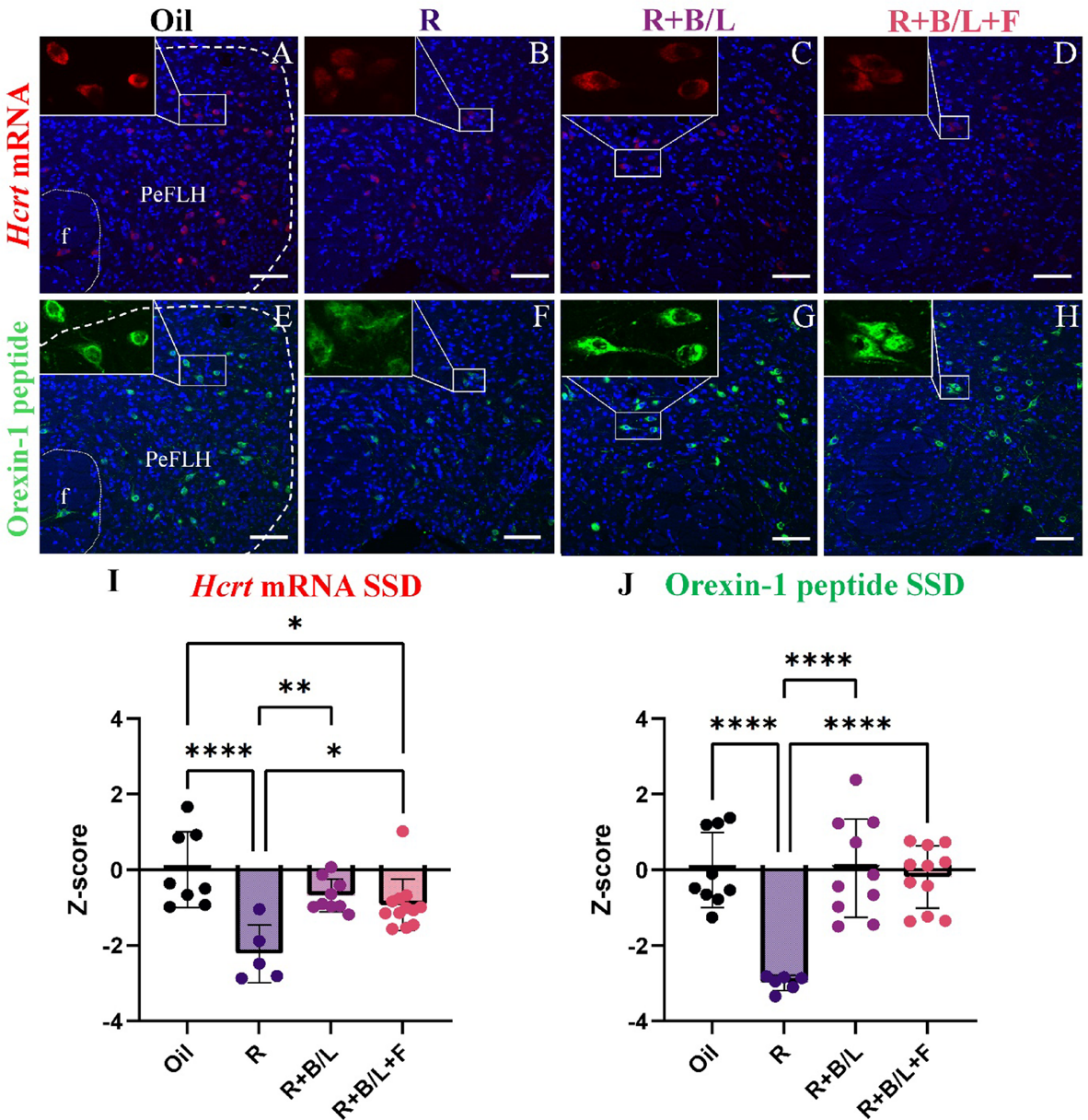
Hypocretin mRNA (*Hcrt*) expression and orexin-1 peptide immunoreactivity in the perifornical region of the lateral hypothalamus (PeFLH) in the rotenone model. Representative images show *Hcrt* mRNA (**A**-**D**, in red) expression and orexin-1 peptide (**E**-**H**, green) immunofluorescence in vehicle (**A** and **D**: oil), (**B** and **F**) rotenone (R)-injected rats. Panel C and G represent a R-treated rat that received benserazide/levodopa therapy also (R + B/L). Panel D and H show representative images from a rat specimen that received besides R + B/L therapy fluoxetine injections also (R + B/L + F). In panels A-H blue color refers to 4′,6-diamidino-2-phenylindole (DAPI) nuclear counterstaining. Scale bar: 50 μm. Panel (**I**) illustrates the comparison of *Hcrt* mRNA specific signal density (SSD) in the PeFLH. Histogram (**J**) provides the comparison of orexin-1 immunoreactivity in the cytoplasm of neurons of the PeFLH. *p < 0.05; **p < 0.01; ****p < 0.0001; *n* = 5–9; one-way ANOVA with Tukey’s *post hoc* test. f: fornix.

Next, we examined the lateral hypothalamic area, where in the perifornical region the orexin-expressing neurons were visualized by RNAscope in situ hybridization for *Hcrt* mRNA combined with immunofluorescence for orexin-1 peptide. As expected, we saw intense cytoplasmic *Hcrt* mRNA signal in full co-localization with strong orexin-1 immunosignal in the same somata. The cell counting revealed that neither the rotenone treatment nor the medication influenced the number of *Hcrt* mRNA (Fig. 3A-D, I, ANOVA: F (3,32) = 0.8483; *p* = 0.4778,) and orexin-1 immunoreactive neurons (Fig. 3E-H, J, ANOVA: F (3,33) = 0.8172; *p* = 0.4936).

In contrast, the *Hcrt* mRNA density was affected by the treatment (Fig. 3A-D, I, ANOVA: F (3,32) = 9.7342; *p* = 0.0002). The amount of *Hcrt* mRNA was drastically reduced in the neurotoxin-treated group (Fig. 3A, B, I, oil vs. R Tukey’s *post hoc* test: *p* = 0.0002) compared to the oil-treated group. Importantly, the B/L antiparkinsonian therapy (Fig. 3B, C, R vs. R + B/L, Tukey’s *post hoc*: *p* = 0.0037) and its combination with fluoxetine (Fig. 3B, D, I, R vs. R + B/L + F, Tukey’s *post hoc*: *p* = 0.0413) significantly improved this effect of the rotenone treatment, but the *Hcrt* mRNA expression remained lower than we observed in control rats suggesting the limited efficacy of B/L and B/L + F therapy on *Hcrt* mRNA expression in Parkinsonian rats (Fig. 3A-D, I). Interestingly, we found a correlation between *Hcrt* mRNA expression and the animals’ motor performance in the rotarod test (Spearman test: ρ = 0,49; *p* = 0.023).

**Fig. 4:**
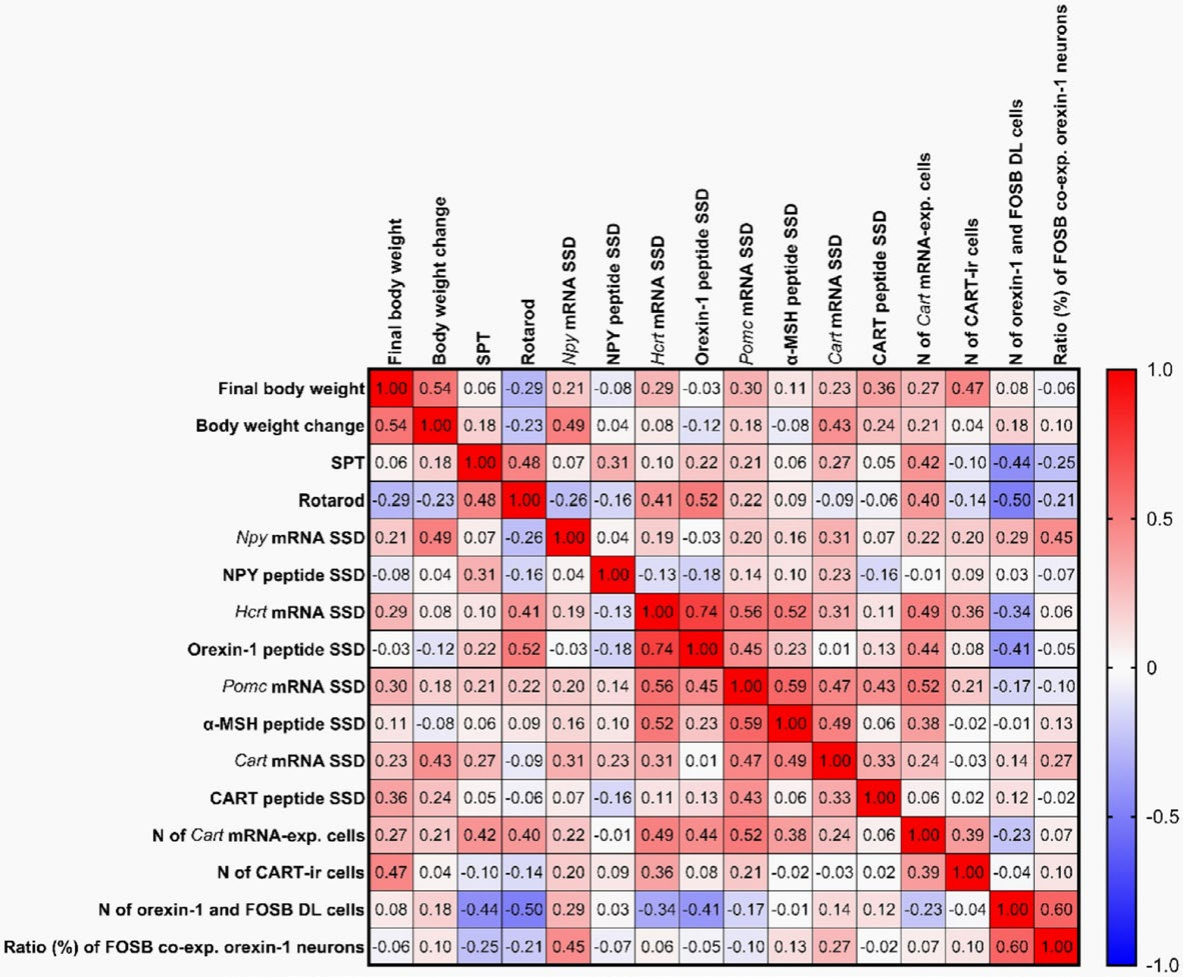
Spearman rank correlation heat map illustrating the relationships between body weight parameters, behavioral test outcomes, and hypothalamic neuropeptide expression across all experimental animals. Color intensity reflects the strength and direction of correlations, with red indicating positive and blue indicating negative associations. Body weight parameters include final body weight and weight change during the experimental period. Behavioral measures comprise the sucrose preference and rotarod tests. Histological parameters include neuropeptide density and mRNA expression levels of NPY, α-MSH, and CART in the ARC, as well as orexin-1 in the LH. Correlation coefficients are presented as Spearman’s ρ values, while exact p-values are provided in Supplementary Table 2.

At the peptide level, the treatment also affected orexin-1 (Fig. 3E- H, J, ANOVA: F (3,32) = 15.33; *p* = 0.0001). In line with our observations at *Hcrt* mRNA level, the orexin-1 SSD decreased following rotenone treatment (Fig. 3E, F, J, oil vs. R, Tukey’s *post hoc*: *p* = 0.0001). Both B/L (Fig. 3F, G, J, R vs. R + B/L, Tukey’s *post hoc*: *p* = 0.9996) and B/L + F (Fig. 3F, H, J, R vs. R + B/L + F, Tukey’s *post hoc*: *p* = 0.9708) therapy restored the orexin-1 peptide levels in the somata of neurons. We saw that the orexin-1 peptide SSD correlated with the rotarod performance (Spearman test: ρ = 0,52; *p* = 0.002) (Fig. 4).

**Fig. 5:**
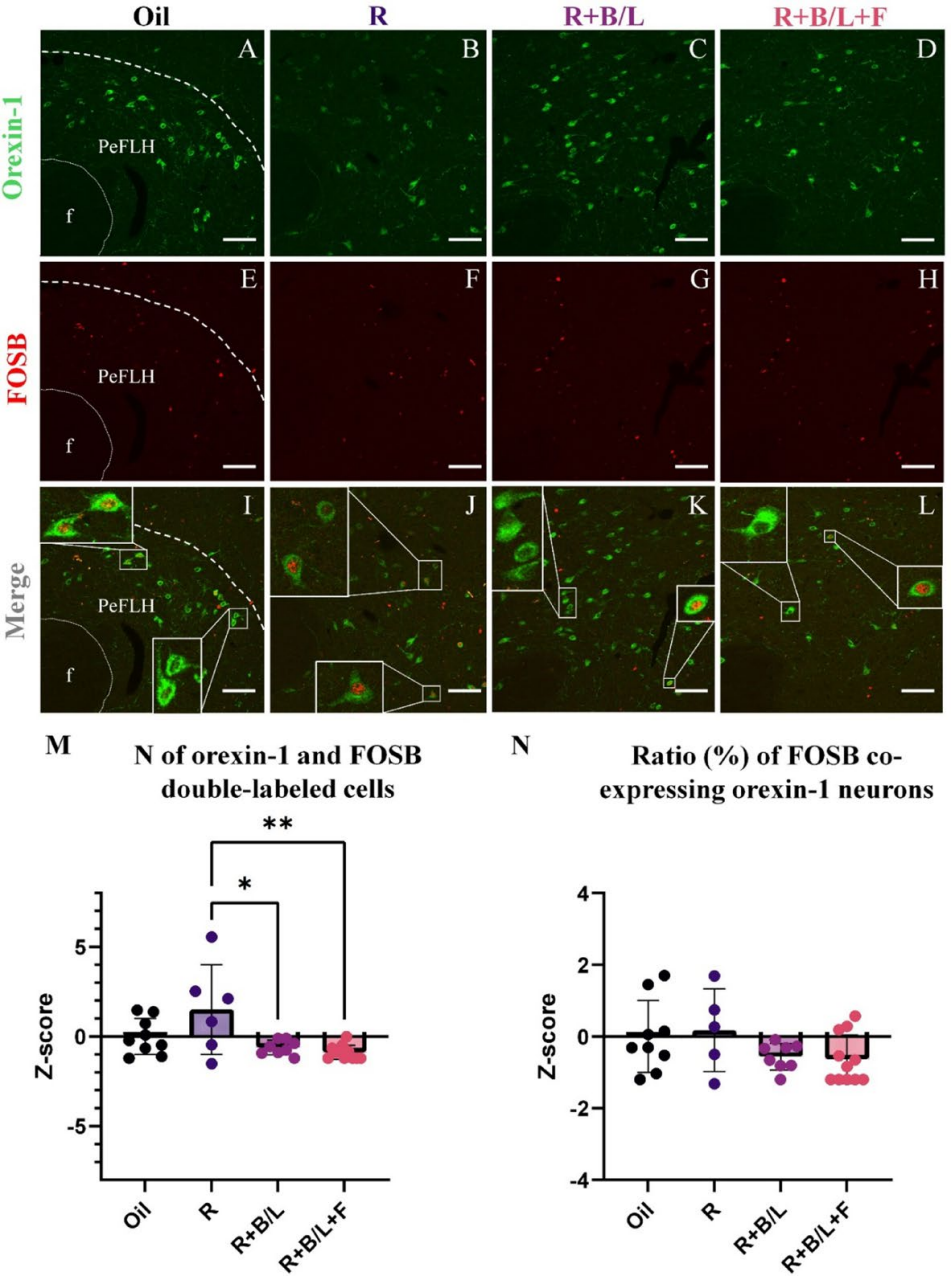
Orexin-1 peptide and FOSB immunoreactivity in the perifornical region of the lateral hypothalamus (PeFLH) in the rotenone model. Representative images show orexin-1 peptide (**A**-**D**, in green), FOSB peptide (**E**-**H**, red) immunofluorescence and the merged image of the two channels (**I**-**L**) in vehicle (**A**, **E**, and **I**: oil), (**B**, **F** and **J**) rotenone (R)-injected rats. Panel (**C**), (**G**) and (**K**) represent a R-treated rat that received benserazide/levodopa therapy also (R + B/L). Panel (**D**), (**H**) and (**L**) show representative images from a rat specimen that received besides R + B/L therapy fluoxetine injections also (R + B/L + F). Scale bar: 50 μm. Panel (**M**) illustrates the number of orexin-1 and FOSB double-labeled cells in the lateral hypothalamic (LH) region. Histogram (**N**) depicts the ratio of orexin-1 cells that were also positive for FOSB in the LH region *p < 0.05; **p < 0.01; *n* = 5–11; one-way ANOVA with Tukey’s *post hoc* test. f: fornix.

In order to demonstrate that orexin-1 cells’ neuronal activity was affected in the model, we performed an orexin-1/FOSB double-labeling. We observed that the treatment affected the chronic activity of orexin-1 neurons (Fig. 5M, ANOVA: F (3,32) = 5.796; *p* = 0.0030). Rotenone induced a moderate rise that was statistically not significant (Fig. 5J, Tukey’s *post hoc*, *p* = 0.0937), however, the B/L (Fig. 5G, M, Tukey’s *post hoc*: *p* = 0.0118) and B/L + F therapy (Fig. 5L, M, *p* = 0.0022) caused a reduction in the activity compared to the neurotoxin-treated group. Interestingly, we saw that the count of orexin-1/FOSB double-labeled cells exerted an inverse correlation with the performance in sucrose preference (Spearman test: ρ=-0,44; *p* = 0.027) and rotarod tests (Spearman test: ρ=-0,50; *p* = 0.004, Fig. 4, Suppl. Table 2). Nevertheless, it has to be stated that the proportion of FOSB positive orexinergic cells was relatively low (Fig. 5I-L). Although there was a statistically significant change in the number of double-labelled cells, the overall proportion of activated cells remained low and did not differ significantly across treatment conditions. In a pilot assessment, we examined the FOSB immunoreactivity in the ARC also. Because we observed very low or negligible neuronal activity in all experimental groups, we did not perform double labelings to assess the activity of peptidergic cells in the ARC.

**Fig. 6:**
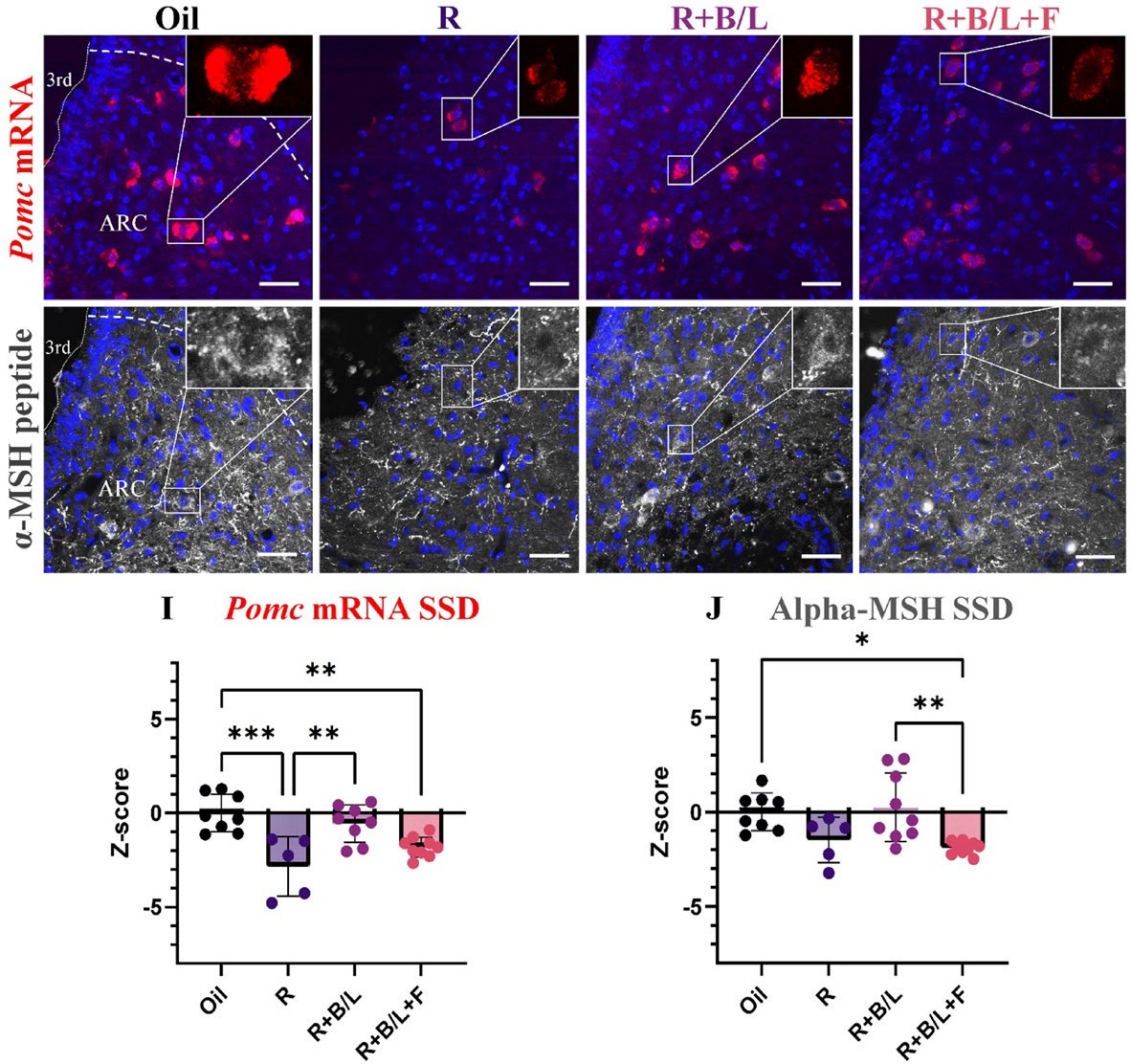
Proopiomelanocortin mRNA (*Pomc*) expression and α–melanocyte-stimulating hormone (α-MSH) peptide immunoreactivity in the arcuate nucleus (ARC) in the rotenone model. Representative images show *Pomc* mRNA (**A**-**D**, in red) expression and α-MSH peptide (**E**-**H**, white) immunofluorescence in vehicle (**A** and **D**: oil) and (**B** and **F**) rotenone (R)-injected rats. Panel (**C**) and (**G**) represent a R-treated rat that received benserazide/levodopa therapy also (R + B/L). Panel (**D**) and (**H**) show representative images from a sample of a rat that received besides R + B/L therapy fluoxetine injections also (R + B/L + F). In panels (**A**)-(**H**) blue color refers to 4′,6-diamidino-2-phenylindole (DAPI) nuclear counterstaining. Scale bar: 50 μm. Panel (**I**) illustrates the comparison of *Pomc* mRNA specific signal density (SSD) in the ARC. Histogram (**J)** provides the comparison of α-MSH immunoreactivity in the cytoplasm of nerve cells of the ARC. *p < 0.05; *p < 0.05; **p < 0.01; ***p < 0.001; *n* = 5–9; one-way ANOVA with Tukey’s *post hoc* test. 3rd: third ventricle.

Next, we examined the anorexigenic neurons of the ARC and performed in situ hybridization for *Pomc* mRNA, combined with immunofluorescence for alpha-MSH. Again, we tested whether the Parkinsonian-like state caused by rotenone-treatment or the therapy affects these neurons. We successfully combined the techniques and detected a relatively strong *Pomc* mRNA expression in the cytoplasm of neurons located in the lateral part of the ARC. We also saw a relatively weak alpha-MSH immunofluorescence in the same perikarya, beyond a more intense immunosignal localized to nerve fibers in the neuropil of the ARC (Fig. 6) No significant change was observable in the rotenone model in the number of POMC neurons among the four groups (Fig. 6A-D, ANOVA: F (3,27) = 2.636; *p* = 0.0700).

ANOVA revealed the main effect of treatment on the *Pomc* mRNA expression in the ARC significant (Fig. 6A-D, I, ANOVA: F (3,26) = 10.3599; *p* = 0.0001). Rotenone treatment reduced *Pomc* expression (Fig. 6A, B, I, oil vs. R, Tukey’s *post hoc*: *p* = 0.0002). Interestingly, the B/L therapy (Fig. 6B, C, I, R vs. R + B/L, Tukey’s *post hoc*: *p* = 0.0027) alone reversed this effect, while it remained ineffective when fluoxetine was also injected (Fig. 6B, D, I, R vs. R + B/L + F, Tukey’s *post hoc*: *p* = 0.2817). At peptide level, we identified similar dynamics (Fig. 6E-H, J, ANOVA: F (3,27) = 6.287; *p* = 0.0022), however the rotenone-induced decline in the alpha-MSH peptide content of the ARC neurons was not significant (Fig. 6E, F, J, Tukey’s *post hoc*: *p* = 0.1643). Again, the B/L therapy appeared to be effective, while in combination with fluoxetine treatment the SSD of alpha-MSH remained low (Fig. 6G, H, I, Tukey’s *post hoc* test, R + B/L vs. R + B/L + F, Tukey’s *post hoc*: *p* = 0.0047).

**Fig. 7:**
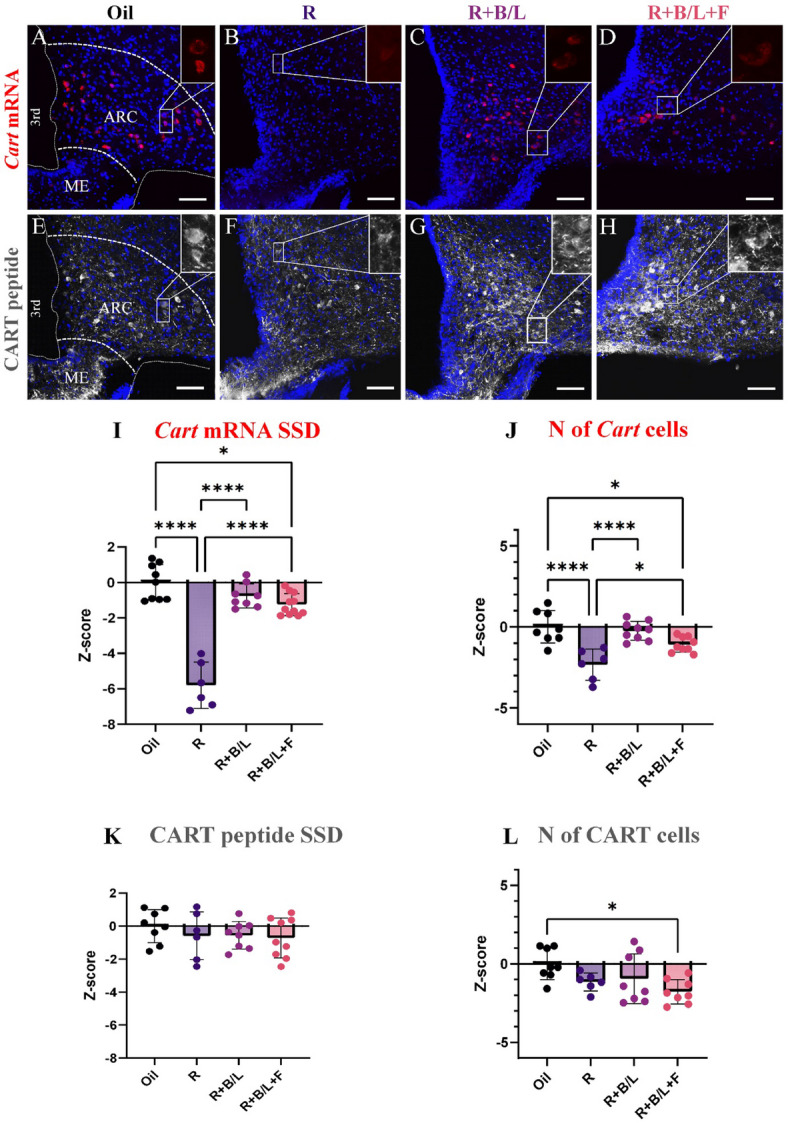
Cocaine-and amphetamine-regulated transcript mRNA (*Cart)* expression and CART peptide immunoreactivity in the arcuate nucleus (ARC) in the rotenone model. Representative images show *Cart* mRNA (**A**-**D**, in red) expression and CART peptide (**E**-**H**, in white) immunofluorescence in vehicle (**A** and **D**: oil) and (**B** and **F**) rotenone (R)-injected rats. Panel (**C**) and (**G**) represent a R-treated rat that received benserazide/levodopa therapy also (R + B/L). Panel (**D**) and (**H**) show representative images from a sample of a rat that received besides R + B/L therapy fluoxetine injections also (R + B/L + F). In panels (**A**)-(**H**) blue color refers to 4′,6-diamidino-2-phenylindole (DAPI) nuclear counterstaining. Scale bar: 50 μm. Panel (**I**) illustrates a comparison of *Cart* mRNA-specific signal density (SSD) in the ARC. Diagram (**J**) presents the number of *Cart* mRNA-expressing cells within the ARC. Panel (**K**) illustrates the magnitude of CART immunoreactivity in the cytoplasm of ARC neurons. Graph (**L**) depicts the number of CART peptide-expressing cells the in the ARC. *p < 0.05; ****p < 0.0001 *n* = 6–11; one-way ANOVA with Tukey’s *post hoc* test. 3rd: third ventricle; ME: median eminence.

Since POMC cells in the ARC co-express CART, we decided to examine this mediator both at mRNA and peptide level in this model. Our RNAscope in situ hybridization combined with immunofluorescence reliably detected cells that contained both *Cart* mRNA signal and CART peptide immunoreactivity in their cytoplasm. We performed the cell counting, and to our surprise, we observed that the number of *Cart* mRNA positive cells decreased upon rotenone treatment (Fig. 7A-D, J, ANOVA: F (3,28) = 13.17; *p* < 0.0001, Tukey’s *post hoc*: *p* < 0.0001) and the reduction was reversed by B/L (Fig. 7B-C, J, R vs. R + B/L, Tukey’s *post hoc*: *p* < 0.0001) therapy. Again, the B/L in combination with fluoxetine exerted a limited efficacy (Fig. 7B, D, J, R vs. R + B/L + F, Tukey’s *post hoc*: *p* = 0.0212). Interestingly, the *Cart* mRNA expression correlated with the body weight change in our rats (Spearman’s test ρ = 0.43; *p* = 0.009, Fig. 4 and Suppl. Table 2).

Regarding the number of CART peptide immunoreactive cells, we observed a reduction of limited magnitude in the combination therapy group (Fig. 7E, H, Fig. 7L, ANOVA: F (3,26) = 3.654; *p* = 0.0254, Tukey’s *post hoc*: *p* = 0.0151). The *Cart* mRNA expression was drastically affected by the treatment (Fig. 7A-D, I, ANOVA: F (3,26) = 57.6051; *p* = 0.0001). Rotenone injections caused a strong downregulation of *Cart* mRNA in the ARC (Tukey’s *post hoc*: *p* = 0.0002). B/L administration resulted in a significant improvement (Fig. 7B-C, I, R vs. R + B/L, Tukey’s *post hoc*: *p* < 0.0001). However, when fluoxetine was also administered, despite a significant improvement (Fig. 7C, I, R vs. R + B/L + F, Tukey’s *post hoc*: p = *p* < 0.0001) the *Cart* mRNA signal still remained significantly below the control value (Fig. 7A, D, I, Oil vs. R + B/L + F, Tukey’s *post hoc*: *p* = 0.019). In contrast to the changes in the *Cart* mRNA expression, no remarkable change occurred in the CART peptide content of ARC neurons (Fig. 7E-H, chart 7 K, ANOVA: F (3.27) = 0.6441; *p* = 0.5934). Although the B/L therapy used (Fig. 7F-G, chart 7 K, R vs. R + B/L, Tukey’s *post hoc*: *p* = 0.0002) and the therapy combined with fluoxetine (Fig. 7F-H, K, R vs. R + B/L + F, Tukey’s *post hoc*: *p* = 0.0002) did not result in significant changes in peptide levels.

## Discussion

In this work we aimed at characterizing how the hypothalamic expression and immunoreactivity of *Npy*/NPY, *Pomc*/α-MSH, *Cart*/CART, and *Hcrt*/orexin-1 in the rotenone model of PD. We successfully tested the hypothesis that the PD-like state disrupts the function of these neuropeptidergic systems, and pharmacotherapy provides only partial reversal of these alterations.

In our study, we applied the rotenone model of PD because, in addition to replicating the motor symptoms of the disease, it also presents non-motor features such as anxiety, depression, and body weight changes^31,33,45,46^. Rotenone primarily targets mitochondrial complex I, impairing energy metabolism in cells with high energy demands, particularly those in the central nervous system, as well as in peripheral organs^45^, such as myocardial tissue^47^ and bone marrow^48^. Importantly, rotenone reproduces key neuromorphological hallmarks of the disorder, including dopaminergic degeneration of the nigrostriatal pathway and the formation of Lewy bodies due to alpha-synuclein accumulation^31,49^. An additional advantage of the model is that rotenone is lipophilic, accumulates in the adipose tissue from where it is gradually released exerting a long-term effect, a feature that mirrors the chronic dynamics of neurodegeneration in PD^50^. As to the peripheral cites of action, it is important to note that no cardiovascular side effects were observed at the dose we used in our study. This is supported by our findings in the rotarod test, because running on the rotating rod implies considerably increased cardiovascular performance. In rotenone-treated animals that received subsequent anti-Parkinson medication, we did not detect compromised performance. Although we cannot rule out that rotenone per se caused some damage in multiple organ systems (e.g. gastrointestinal system, kidney, bone marrow, skeletal muscles) that might have influenced our results, we did all efforts to minimize these by setting the lowest effective dose very carefully and individually (^31,33^).

To assess mood-related changes, we employed the sucrose preference test (SPT). We decided for SPT because deteriorated motor skills limit the reliability of this paradigm to a lesser extent than that of other commonly used behavioral assays for the assessment of motor skills, such as the forced swim, open field, or light–dark box tests^31,51^. Nevertheless, we have to state that the deterioration of motor skills may affect the reliability of all behavioral tests in animal models because the animal’s performance is ultimately the result of executive motor systems. However, in our view, reduced motor performance has the least impact on the outcome of the SPT as supported by unaltered total fluid consumption in rotenone-treated rats (Suppl. Fig. 2).

Over the 6-week study period, the body weight of the rotenone-treated groups declined significantly compared to controls, and this effect was not mitigated by pharmacological interventions. We also observed direct correlations between the bodyweight change and *Npy* mRNA moreover *Cart* mRNA expressions (Fig. 4) suggesting link between our morphological results and the body weight change. These findings are comparable with observations in human PD, where marked reductions in body mass index and body fat have been reported^52,53^. Importantly, neither levodopa^54^ nor fluoxetine therapy^55^ have been shown to reverse this weight loss.

Our findings demonstrate that rotenone treatment caused motor coordination deficits in our animals, which were alleviated by anti-Parkinson medication. Furthermore, we observed an increase in anhedonia, which was not significantly improved by B/L therapy alone, but was effectively reversed when combined with fluoxetine. These results confirm the validity of the rotenone model in our hands and strongly suggest that the functional-morphological changes in the hypothalamic orexigenic and anorexigenic peptidergic systems discussed below are associated with the PD-like state and therapeutic effects. Our correlation analyses (Fig. 4) suggest that alterations in orexinergic cells influence both the motor performance, and the mood status.

Our findings revealed that the somata of neurons in the ARC contained mostly both the precursor mRNAs and the corresponding neuropeptide protein products. In case of NPY, however, the *Npy* mRNA was localized to the perikarya, our antibody did not detect the NPY peptide within the cell bodies. Instead, it revealed a dense network of immunoreactive axons in the ARC neuropil, which is consistent with previous findings by our^39^ and other^41^ research groups. This observation can be attributed to the fact that however peptide synthesis takes place on ribosomes in the perikaryon, the NPY peptide product is quickly transported through the axon towards the terminals. Therefore, the detection of the NPY neuropeptide in the somata by immunofluorescence without inhibiting the trafficking, for example by intracerebroventricular colchicine treatment, is not possible^39^. Considering the deleterious effects of colchicine that affects both general condition and behavior of animals, its application was not adjustable with the goal of this study in this experimental setup. Therefore, we decided to perform the semi-quantitation of NPY immunosignal in the neuropil of the ARC.

In the ARC, despite a correlation between Npy mRNA and bodywight data, we did not find a significant change in either *Npy* mRNA or NPY peptide levels upon rotenone treatment, suggesting that ARC/NPY does not play a crucial role in the development of PD-associated body weight changes. Literature data are not consistent in this question: increased *Npy* mRNA expression was found in caudate nucleus, putamen, and nucleus accumbens samples obtained from brains of PD patients^56^. In contrast, reduced NPY peptide level was found in Parkinsonian cerebrospinal fluid (CSF) samples^57^. Similar findings were observed in CSF samples from patients with major depressive disorder (MDD)^58^. Inconsistent findings are likely to be explained by the differences in the sample sources: here we examined the ARC, while other studies focused on the basal ganglia or CSF.

Anti-Parkinson therapy resulted in moderate reduction of *Npy* mRNA expression, whereas in combination with fluoxetine it did not yield significant changes in NPY at either the mRNA or peptide level. The effect of B/L therapy used in PD on NPY production in the ARC region has not been previously investigated. However, levodopa has previously been reported to moderately increase NPY peptide levels in the striatum in the 6-hydroxydopamine (6-OHDA) rat model of PD^45^, and also in human samples^59^. By contrast, it has been reported in rats that long-term fluoxetine treatment alone increases *Npy* mRNA expression in the ARC^60^. These findings indicate that levodopa exerts region-specific effects, increasing *Npy* expression in the striatum while slightly reducing it in the ARC. Notably, when combined with fluoxetine, this treatment reverses the levodopa-induced decrease in *Npy* mRNA levels within the ARC.

A significant decrease in *Hcrt* mRNA and orexin-1 peptide density was observed in the LH region in the rotenone-treated group without significant reduction of orexinergic cell count. On one hand, this partially contradicts human studies where selective orexinergic neuron loss was described in PD patients. This discrepancy might be explained by the decrease of orexinergic neuron counts in the hypothalamus^61^ in old age since our rats were middle-aged (8-months-old) when the brains were collected. On the other hand, the results on reduced orexin-1 mRNA and peptide level in our Parkinsonian rats fit well with lower orexin-1 levels described in postmortem prefrontal cortex^62^ and cerebrospinal fluid^63^ samples from patients with PD and MDD^64^. These findings ultimately suggest that decreased orexin production in the LH may contribute significantly to the energy imbalance, weight loss and somnolence in PD.

We found no significant difference in the relative ratio of FOSB and orexin-1 between the groups, moreover, the low proportion of FOSB immunoreactive orexin-1 neurons suggests that the rotenone-induced PD-like state does not evoke transcription of new genes in orexinergic neurons of the LH via activator protein 1^65^.

Our observation that anti-Parkinson therapy, both alone and in combination, reversed the neurotoxin-induced decrease in *Hcrt*/orexin-1 in the view that fluoxetine does not significantly affect orexin-1 production in the LH^66^ suggests that levodopa may have a previously unrecognized effect of increasing orexin-1 levels.

A limited amount of data has accumulated on hypothalamic α-MSH and CART in PD. The mitochondrial antioxidant features have been recognized^67,68^. In the 1-methyl-4-phenyl-1,2,3,6-tetrahydropyridine (MPTP) model of PD, in M17 neuroblastoma cells, α-MSH pretreatment reduced dopaminergic cell damage, and similar protective effects were observed upon CART pretreatment in the MPTP model of PD in mice^69,70^. Additionally, in mouse models, significant improvement occurred both at the behavioral level and in motor coordination^67^. These studies suggested the potential therapeutic significance of CART and α-MSH in PD^67,68^.

In the ARC region, we detected a reduction in both *Pomc* and *Cart* mRNA density in the neurotoxin-treated group, while the peptide content remained unchanged. We propose that while peptide synthesis was diminished, the energy deficit induced by rotenone impaired the neurons’ ability to transport α-MSH and CART to the terminals. Consequently, they accumulated within the neuronal cytoplasm. The lack of α-MSH^68^ and CART^67^ to exert their antioxidant effects likely contributed to disease progression. Moreover, this lower anorexigenic activity may explain the weight gain observed in certain cases of PD. Regarding the possible site of action, CART may also have been unable to mediate its inhibitory influence on food intake in the area postrema^15^ or α-MSH may not exert its inhibitory effect in the LH or paraventricular nucleus of the hypothalamus^69^, ultimately promoting increased feeding.

We observed that, despite the reduced mRNA levels, peptide levels remained unchanged. This is likely due to an energy deficit. Rotenone on one hand disrupts the mitochondria at complex I, causing a decrease in energy levels, which in turn disrupts the axonal transport of peptides, on the other hand, disturbed microtubular function may also disturb the trafficking^70,71^. Consequently, although mRNA production decreases, the peptide may accumulate in the cell’s soma^31^.

With anti-Parkinson therapy, *Pomc*/α-MSH levels normalized, while the antidepressant-combined treatment did not affect the reduced neuropeptide expression. Previous mouse experiments have shown that long-term administration of fluoxetine reduced 5-hydroxytryptamine 2 C receptor expression and signaling, thereby downregulating *Pomc* expression and α-MSH levels in hypothalamic neurons^72^.

As to *Cart*/CART, both therapeutic approaches reversed the rotenone-induced reduction. This is in line with an earlier study using the 6-OHDA model, where levodopa restored diminished CART levels^73^. This observation suggests that levodopa therapy may have a weight-gaining effect by enhancing appetite and increasing food intake in some cases of PD. This is supported also by our finding that the *Cart* mRNA level correlated with the bodyweight data (Fig. 4).

In our recent studies we found neurodegeneration of urocortin 1-expressing cells in the centrally projecting Edinger-Westphal nucleus in the rotenone model^31,33^. Therefore, in this study we also have counted the hypothalamic peptidergic cells. The number of *Npy*/NPY, *Pomc*/α-MSH and *Hcrt*/orexin-1 mRNA/peptide-containing cells were not affected by rotenone treatment in the present study, suggesting that they do not suffer neurodegeneration. We only saw a limited reduction of *Cart* mRNA-expressing cell numbers. Considering that we did not see any changes in the *Pomc*/α-MSH cell count and *Pomc* and *Cart* are co-expressed in the ARC^74^, we assume that the marked downregulation of *Cart* mRNA expression explains the lower *Cart* cell counts in the ARC, rather than neurodegeneration.

Nevertheless, our study has certain limitations, which are discussed below. The regulation of energy homeostasis is an extremely complex process that recruits hundreds of messengers^75^. This study was focused on the hypothalamic peptidergic key regulators. Nevertheless, we must acknowledge that several other neural circuits may also contribute to PD. For instance, the examination of the recruitment of Agouti-related peptide^76^, melanin-concentrating hormone^77^, cholecystokinin^78^, glucagon-like peptide-1^79^, and leptin remain for future studies in the present and also in other models of PD.

Another actual limitation of this work is that we did not include female rats in our study. Therefore, we don’t know whether sex-differences exist in the recruitment of the examined hypothalamic neuropeptides in the rotenone model of PD. We decided not to include female rats in this experiment because of the following uncontrollable circumstances. On one hand, the sex hormone sensitivity of hypothalamic centers is well known, as the existence of estrogen receptors in several hypothalamic regions, including the ARC^80^, and LH^81^ has been described. In the medial region of the ARC, estrogen receptors are expressed both in *Pomc*^82^ and *Npy*-producing neurons^83^. Similarly, orexin-1-producing cells of LH are also estrogen sensitive modulating regulation of food intake and reproduction^84,85^. This strongly suggests that the hormonal fluctuations related to the estrous cycle could interfere with the functional activity of these peptidergic systems and this could also interact with response to rotenone or pharmacotherapy. On the other hand, we also predict that rotenone administration per se may disturb the estrus cycle as it causes a chronic stress state^86^ and the effect of chronic mild stress was shown to deteriorate the regularity of the estrus cycles in rats^87^. Therefore, carefully planned and controlled future experiments will be required to examine female animals in this context and model.

It is also important to claim, that the effect of anti-Parkinsonian therapy and the fluoxetine treatment had most likely pleiotropic effects in the brain. Considering the wide afferent connectivity of the ARC^88^ and LH area^89^ it is possible that the hypothalamic functional-morphological alterations observed here are in part attributable to the indirect effects, for instance via brain areas influenced by dopaminergic and serotonergic afferents. We also have to state the relative inefficacy of our treatments on peptidergic changes was observed in a six-week model, where we applied 3 weeks pharmacotherapy. A longer treatment period, higher dose or a different way of administration may have had a different influence.

Finally, in this study we were unable to conduct individual food intake measurements that could provide valuable data regarding energy homeostasis. We decided to abandon these measurements because this would have required continuous social isolation of animals, and this is a strong stress factor^90^ that could have significantly altered the levels of these stress-sensitive hypothalamic neuropeptides^91,92,93^.

## Conclusions

With respect to the limitations, we described the dynamics of four cardinal neuropeptides controlling the energy metabolism in the PD rotenone model in male rats both at mRNA and peptide levels. We observed that following rotenone treatment, orexin-1 and α-MSH levels decreased both at the mRNA and peptide level, whereas for NPY and CART a reduction was seen only for the mRNAs, but not at peptide level. *Npy* and *Cart* mRNAs were in correlation with the body weight change, while the mood state changes of our rats correlated with the alterations in the orexinergic neurons. B/L monotherapy and combined treatment with fluoxetine resulted in reversal of rotenone-induced reduction in NPY, orexin-1, and CART expression at both the mRNA and peptide levels. In contrast, for α-MSH, B/L therapy was associated with restored neuropeptide expression at both levels. In animals subjected to combination therapy, the elevation in *Pomc* mRNA expression was not paralleled by a corresponding α-MSH increase. Our findings indicate complex functional alterations in both the orexinergic and anorexigenic systems that are not effectively alleviated by B/L or B/L + F therapy. Future human studies have to determine how these changes contribute to weight gain or weight loss in PD^8^ and may help to find new individualized therapeutic interventions to alleviate disturbances of energy homeostasis in PD.

## Supplementary Information

Below is the link to the electronic supplementary material.

Supplementary Material 1

Supplementary Material 2

Supplementary Material 3

Supplementary Material 4

## Data Availability

All data underlying the results of this study are available from the corresponding author on request.

## Abbreviations

PD: Parkinson’s disease
PBS: Phosphate-buffered physiological saline
PFA: Paraformaldehyde
POMC: Pro-opiomelanocortin
NPY: Neuropeptide Y
ARC: Arcuate nucleus
α–MSH: α–melanocyte-stimulating hormone
CART: Cocaine-and amphetamine-regulated transcript
CRH: Corticotropin-releasing hormone
SPT: Sucrose preference test
RPT: Rotarod test
LH: Lateral hypothalamus
DMSO: Dimetil-sulphoxyde
B/L: Benserazide/levodopa
SSD: Specific signal density
CSF: Cerebrospinal fluid
MDD: Major depressive disorder
6-OHDA: 6-hydroxydopamine
NDS: Normal donkey serum
Cy3: Cyanine 3
DAPI: 4′,6-diamidino-2-phenylindole
MPTP: 1-methyl-4-phenyl-1,2,3,6-tetrahydropyridine
