# Supplementary figures and images for: Hypothalamic orexigenic and anorexigenic neuropeptides in the rotenone model of Parkinson’s disease
Source: Sci Rep. 2026 May 4;16:20607. doi: 10.1038/s41598-026-51774-7 (PMC13333943; doi:10.1038/s41598-026-51774-7)

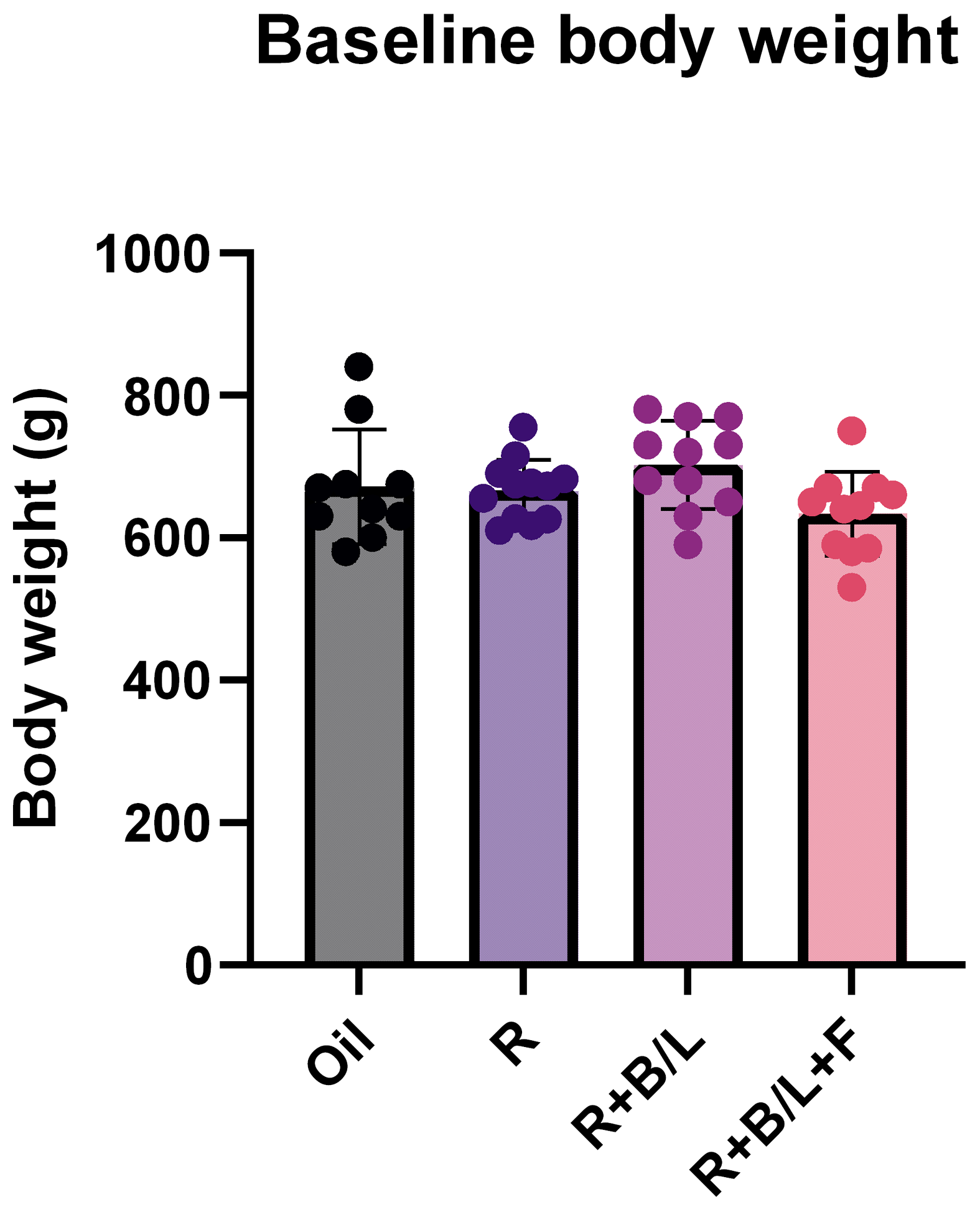

Supplement: Supplementary file 1 — Supplementary Material 1 [file 41598_2026_51774_MOESM1_ESM.tif]

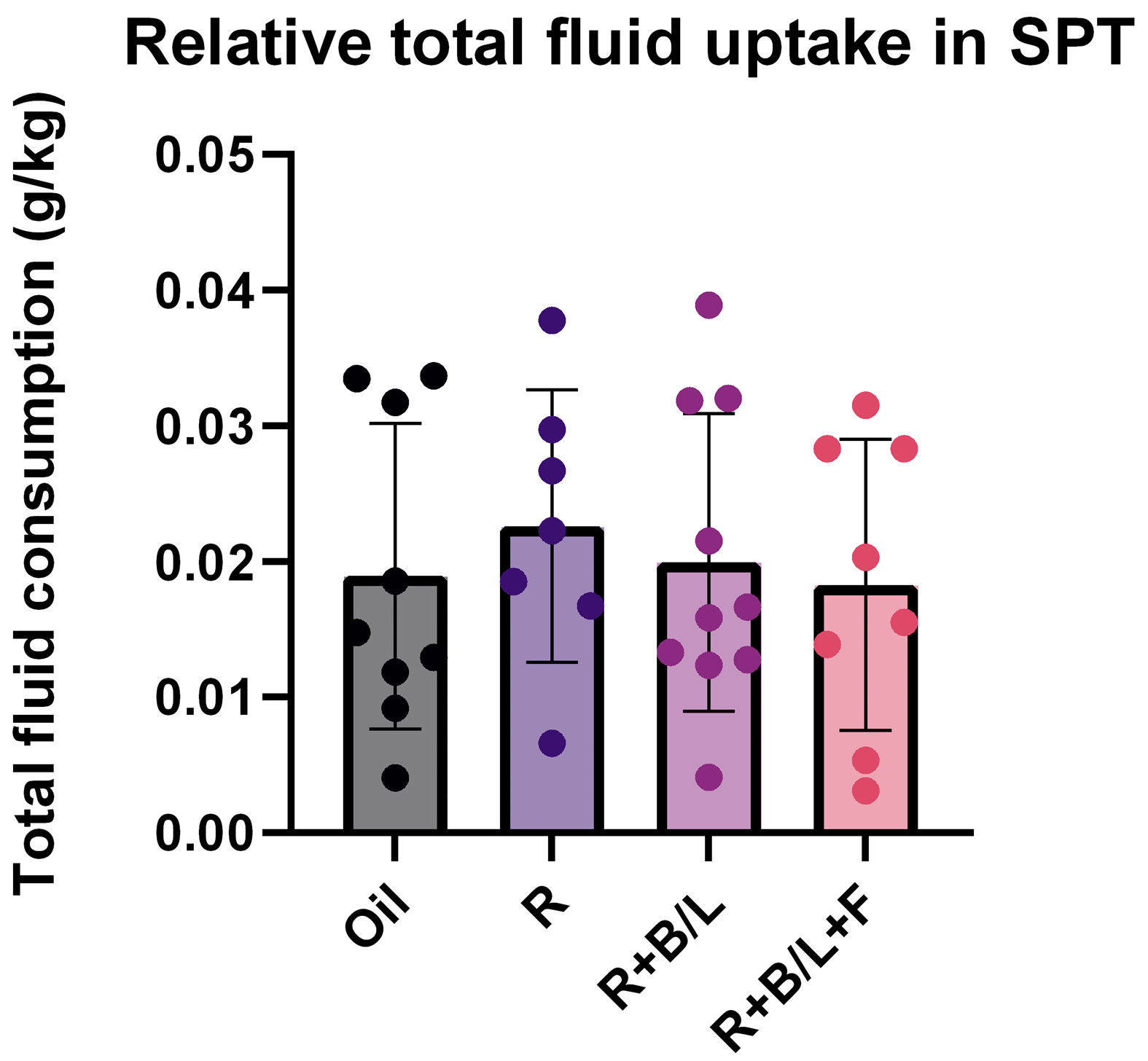

Supplement: Supplementary file 2 — Supplementary Material 2 [file 41598_2026_51774_MOESM2_ESM.tif]
