# Supplementary material for: Hypothalamic orexigenic and anorexigenic neuropeptides in the rotenone model of Parkinson’s disease
Source: Sci Rep. 2026 May 4;16:20607. doi: 10.1038/s41598-026-51774-7 (PMC13333943; doi:10.1038/s41598-026-51774-7)
Supplement: Supplementary file 3 — Supplementary Material 3 [file 41598_2026_51774_MOESM3_ESM.docx]

|  | **F** | **p** |
| --- | --- | --- |
| Body Weight Change (%) **week 1** | **6.071** | **0.0017** |
| Body Weight Change (%) **week 2** | **6.198** | **0.0015** |
| Body Weight Change (%) **week 3** | **5.986** | **0.0018** |
| Body Weight Change (%) **week 4** | **9.183** | **<0.0001** |
| Body Weight Change (%) **week 5** | **10.33** | **<0.0001** |
| Body Weight Change (%) **week 6** | **4.86** | **0.0056** |
| ***Npy*** mRNA SSD | **3.36** | **0.0346** |
| **NPY** peptide SSD | 0.4694 | 0.7059 |
| ***Hcrt*** mRNA SSD | **9.735** | **0.0001** |
| **Orexin-1** peptide SSD | **15.33** | **<0.0001** |
| N of **Orexin-1** and **FOSB** double-labeled cells | **5.796** | **0.003** |
| Ratio of **FOSB**-ir **orexin-1** neurons (%) | 1.894 | 0.1527 |
| ***Pomc*** mRNA SSD | **10.36** | **0.0001** |
| **α-MSH** SSD | **6.287** | **0.0022** |
| ***Cart*** mRNA SSD | **57.61** | **<0.0001** |
| **CART** SSD | 0.6441 | 0.5934 |
| N of ***Cart***-expr. cells | **13.17** | **<0.0001** |
| N of **CART**-ir cells | **3.654** | **0.0254** |

**Supplementary table 1:** Summary of F and p values obtained in the statistical evaluation by one-way analysis of variance (ANOVA) tests. Significant values are highlighted in **bold**. SSD: specific signal density.
