# Supplementary material for: Hypothalamic orexigenic and anorexigenic neuropeptides in the rotenone model of Parkinson’s disease
Source: Sci Rep. 2026 May 4;16:20607. doi: 10.1038/s41598-026-51774-7 (PMC13333943; doi:10.1038/s41598-026-51774-7)
Supplement: Supplementary file 4 — Supplementary Material 4 [file 41598_2026_51774_MOESM4_ESM.docx]

|  | **Final body weight (g)** | **Body weight change** | **SPT** | **Rotarod** | *Npy* **mRNA (SSD)** | **NPY peptide (SSD**) | *Hcrt* **mRNA (SSD)** | **Orexin-1 peptide (SSD**) | *Pomc* **mRNA (SSD)** | **α-MSH peptide (SSD)** | *Cart* **mRNA (SSD)** | **CART peptide (SSD**) | **N of** *Cart* **mRNA-exp. cells** | **N of CART-ir cells** | **N of orexin-1 and FOSB DL cells** | **Ratio (%) of FOSB co-exp. orexin-1 neurons** |
| --- | --- | --- | --- | --- | --- | --- | --- | --- | --- | --- | --- | --- | --- | --- | --- | --- |
| **Final body**  **weight (g)** |  | 0,0005 | 0,7680 | 0,1079 | 0,2701 | 0,6406 | 0,1011 | 0,8658 | 0,1081 | 0,5732 | 0,1774 | 0,0465 | 0,1309 | 0,0070 | 0,6506 | 0,7566 |
| **Body weight change** | 0,000536 |  | 0,3603 | 0,2059 | 0,0064 | 0,8376 | 0,6434 | 0,5006 | 0,3401 | 0,6684 | 0,0097 | 0,1862 | 0,2473 | 0,8082 | 0,3073 | 0,5815 |
| **SPT %** | 0,767952 | 0,3603 |  | 0,0072 | 0,7655 | 0,1283 | 0,6394 | 0,2799 | 0,3528 | 0,7739 | 0,1713 | 0,8300 | 0,0372 | 0,6415 | 0,0271 | 0,2309 |
| **Rotarod** | 0,107896 | 0,2059 | 0,0072 |  | 0,1942 | 0,3932 | 0,0232 | 0,0025 | 0,2741 | 0,6468 | 0,6327 | 0,7676 | 0,0358 | 0,4634 | 0,0042 | 0,2577 |
| *Npy* **mRNA (SSD)** | 0,270144 | 0,0064 | 0,7655 | 0,1942 |  | 0,8242 | 0,3415 | 0,8938 | 0,3401 | 0,4549 | 0,1216 | 0,7350 | 0,2852 | 0,3532 | 0,1443 | 0,0198 |
| **NPY peptide (SSD)** | 0,640574 | 0,8376 | 0,1283 | 0,3932 | 0,8242 |  | 0,4861 | 0,3308 | 0,4932 | 0,6097 | 0,2216 | 0,4281 | 0,9754 | 0,6465 | 0,8597 | 0,7221 |
| *Hcrt* **mRNA (SSD)** | 0,101058 | 0,6434 | 0,6394 | 0,0232 | 0,3415 | 0,4861 |  | 0,0000 | 0,0026 | 0,0043 | 0,0823 | 0,5739 | 0,0069 | 0,0552 | 0,0650 | 0,7716 |
| **Orexin-1 peptide (SSD**) | 0,865839 | 0,5006 | 0,2799 | 0,0025 | 0,8938 | 0,3308 | 0,0000 |  | 0,0169 | 0,2353 | 0,9407 | 0,4718 | 0,0132 | 0,6859 | 0,0196 | 0,7759 |
| *Pomc* **mRNA (SSD)** | 0,108069 | 0,3401 | 0,3528 | 0,2741 | 0,3401 | 0,4932 | 0,0026 | 0,0169 |  | 0,0009 | 0,0133 | 0,0399 | 0,0083 | 0,3059 | 0,3987 | 0,6275 |
| **α-MSH peptide (SSD)** | 0,573174 | 0,6684 | 0,7739 | 0,6468 | 0,4549 | 0,6097 | 0,0043 | 0,2353 | 0,0009 |  | 0,0083 | 0,7809 | 0,0644 | 0,9307 | 0,9470 | 0,5241 |
| *Cart* **mRNA (SSD)** | 0,177438 | 0,0097 | 0,1713 | 0,6327 | 0,1216 | 0,2216 | 0,0823 | 0,9407 | 0,0133 | 0,0083 |  | 0,0737 | 0,1873 | 0,8956 | 0,4399 | 0,1389 |
| **CART peptide (SSD)** | 0,04648 | 0,1862 | 0,8300 | 0,7676 | 0,7350 | 0,4281 | 0,5739 | 0,4718 | 0,0399 | 0,7809 | 0,0737 |  | 0,7463 | 0,9112 | 0,5430 | 0,9017 |
| **N of** *Cart* **mRNA-exp. cells** | 0,130913 | 0,2473 | 0,0372 | 0,0358 | 0,2852 | 0,9754 | 0,0069 | 0,0132 | 0,0083 | 0,0644 | 0,1873 | 0,7463 |  | 0,0348 | 0,2435 | 0,7235 |
| **N of CART-ir cells** | 0,007029 | 0,8082 | 0,6415 | 0,4634 | 0,3532 | 0,6465 | 0,0552 | 0,6859 | 0,3059 | 0,9307 | 0,8956 | 0,9112 | 0,0348 |  | 0,8557 | 0,5977 |
| **N of orexin-1 and FOSB DL cells** | 0,65059 | 0,3073 | 0,0271 | 0,0042 | 0,1443 | 0,8597 | 0,0650 | 0,0196 | 0,3987 | 0,9470 | 0,4399 | 0,5430 | 0,2435 | 0,8557 |  | 0,0002 |
| **Ratio (%) of FOSB co-exp.orexin-1 neurons** | 0,756576 | 0,5815 | 0,2309 | 0,2577 | 0,0198 | 0,7221 | 0,7716 | 0,7759 | 0,6275 | 0,5241 | 0,1389 | 0,9017 | 0,7235 | 0,5977 | 0,0002 |  |

**Supplementary Table 2:** Summary of p values of correlation analyses shown in Figure 4.
